# Primary aortoenteric fistula related to septic aortitis

**DOI:** 10.1590/S1516-31802001000400008

**Published:** 2001-07-07

**Authors:** Fábio Lambertini Tozzi, Erasmo Simão da Silva, Fernando Campos, Henrique Oscar de Azevedo Fagundes, Marcos Lucon, Renato Micelli Lupinacci

**Keywords:** Septicaortitis, Primary aortoenteric fistula, Aneurysm, Pseudoaneurysm, Aortite séptica, Fístula aortoenterica primária, Aneurisma, Pseudoaneurisma

## Abstract

**CONTEXT:**

Primary aortoenteric fistulas usually result from erosion of the bowel wall due to an associated abdominal aortic aneurysm. A few patients have been described with other etiologies such as pseudoaneurysm originating from septic aortitis caused by *Salmonella*.

**OBJECTIVE:**

To present a rare clinical case of pseudoaneurysm caused by septic aortitis that evolved into an aortoenteric fistula.

**CASE REPORT:**

A 65-year-old woman was admitted with Salmonella bacteremia that evolved to septic aortitis. An aortic pseudoaneurysm secondary to the aortitis had eroded the transition between duodenum and jejunum, and an aortoenteric fistula was formed. In the operating room, the affected aorta and intestinal area were excised and an intestine-to-intestine anastomosis was performed. The aorta was sutured and an axillofemoral bypass was carried out. In the intensive care unit, the patient had a cardiac arrest that evolved to death.

## RESUMO

**CONTEXTO:**

Na maioria dos casos, as fistulas aortoentericas primárias resultam da erosão da parede do intestino por um aneurisma da aorta abdominal. Poucos pacientes foram descritos com etiologias distintas.

**OBJETIVO:**

O propósito desse trabalho é apresentar uma rara situação clínica, aortite séptica por Salmonella, que evoluiu para a formação de um pseudoaneurisma da aorta que perfurou a transição entre o duodeno e o jejuno.

**RELATO DE CASO:**

Uma paciente de 65 anos, diabética, foi admitida no hospital com bacteremia por Salmonella, que evoluiu para aortite séptica. Um mês após a alta hospitalar a paciente retornou apresentando psedoaneurisma da aorta abdominal infra-renal, que corroeu a transição entre o duodeno e o jejuno originando uma fístula aorto-entérica primária. Foi submetida ao tratamento cirúrgico, sendo realizada ligadura da aorta, remoção do hematoma infectado, enterectomia, êntero-êntero anastomose e enxerto extra-anatômico axilo-bifemoral com prótese de dacron. Na unidade de terapia intensiva a paciente apresentou parada cardiorrespiratória e óbito.

## INTRODUCTION

Aortoenteric fistulas are classified as primary^1^ and secondary,^2^ (after aortic repair by means of an arterial prosthesis). This condition involves arterial rupture and infection of vascular areas.^3^ Primary fistulas are in most cases (90%)^4^ the result of erosion of the bowel wall, caused by abdominal aortic aneurysm. Septic aortitis is also one of the most challenging problems that confront the vascular surgeon. Transient bacteremia allows lodgment of bacteria on the inner arterial surface and permits the formation of an aneurysm or false aneurysm. Primary fistulas can develop as a result of this pathogenic process.^4-9^ The aim of this work is to present the case report of a patient with a pseudoaneurysm due to *Salmonella* aortitis, which originated an aortoenteric fistula.

## CASE REPORT

A 65-year-old black woman with a twenty-year history of diabetes mellitus was admitted with diffuse abdominal pain and fever. The abdomen was distended without palpable abdominal masses. Her blood pressure was 220 × 120 mmHg, temperature 38.2°C, and the white blood cell count was 23,000 leukocytes per mm^3^. Abdominal radiography and ultrasonography were unremarkable. A blood culture grew *Salmonella non-typhymurium*, and she was treated intravenously with ceftriaxone and discharged on the 11^th^ day. Cerriaxone was substitute for oral amoxicillin.

**Figure 1:**
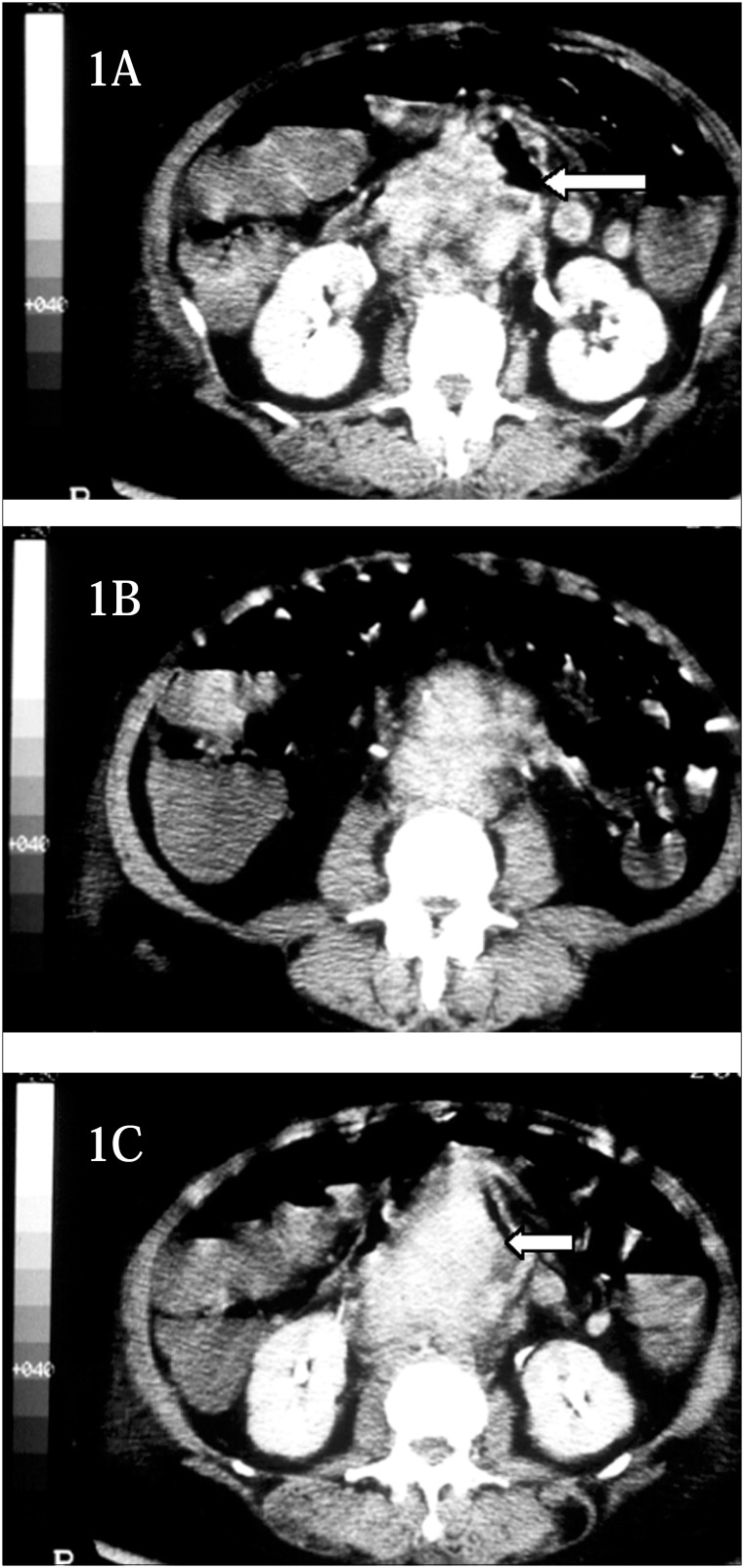
CT scan reveals a large pseudoaneurysm with air close to aortic wall (arrows).

Thirty days after discharge, the patient returned with diffuse abdominal pain, associated with fever (39 °C). Physical examination brought into evidence a pulsatile, epigastric and periumbilical abdominal mass. B-mode ultrasound scanning showed an infrarenal aortic aneurysm, 5.7 cm in diameter. While waiting for abdominal tomography (CT scan), to make an examination of the aortic dilation, the patient had three hematemesis episodes. Endoscopy was repeated three times and only in the last of these was a pulsatile lesion shown in the fourth portion of the duodenum. In order to analyze the anatomy of the aneurysm, an emergency CT scan was performed, which displayed a large pseudoaneurysm with gas close to the arterial wall (Figure 1).

Eighteen hours after the first bleeding, the patient was submitted to a midline laparotomy, which revealed a large retroperitoneal hematoma densely adhering to the duodenum-jejunum transition. After proximal aortic control the fistula was closed off and a large intestinal defect was detected. The affected intestinal area was removed and intestine-to-intestine anastomosis was performed. The infected aorta and the large hematoma were excised, and the proximal aorta and iliac common arteries were oversewn. A right axillobifemoral bypass graft with prosthesis was constructed.

Eight hours after the end of the surgery, the patient had cardiac arrest and was unresponsive to resuscitative maneuvers. Necropsy could not identify the cause of death and a metabolic origin was considered. The infrarenal aortic specimen exhibited the presence of some fatty streaks, but there was no massive atherosclerotic disease.

## DISCUSSION

**Figure 2:**
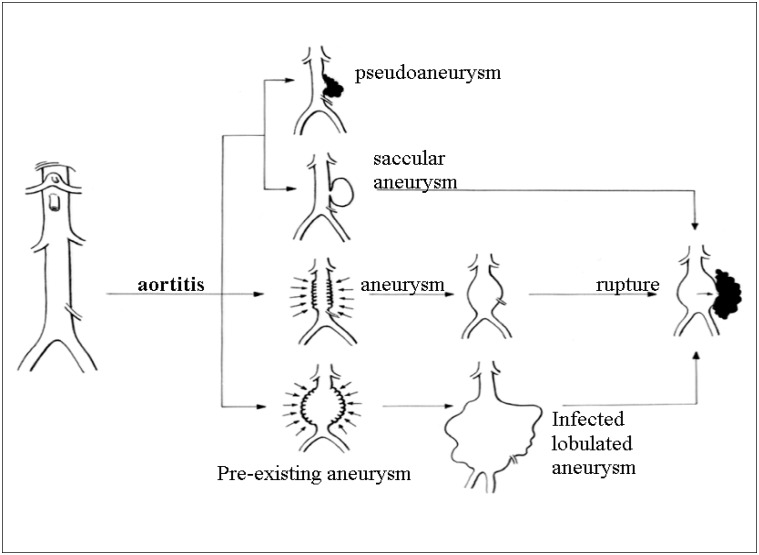
Infectious aortitis could affect a normal aorta or aneurysmal aorta with different forms of presentation.

Classifications used for describing arterial infection include several different names, such as mycotic aneurysm, infected aneurysm, aortitis, cryptogenic aortitis, bacterial aortitis and microbial arteritis.^2^ Microbial arteritis is an infectious process that attacks a non-aneurysmal artery and develops an aneurysm or arterial rupture with pseudoaneurysm^2^ (Figure 2). Our patient presented microbial arteritis caused by Salmonella. Fever, abdominal distension and pain were some of the common aortitis diagnostic findings in our patient. When aortic infection leads to aneurysm or pseudoaneurysm formation, there may be a pulsatile mass present. In the initial phase of the aortitis, there may not be any remarkable findings from either ultrasound or tomography. In our clinical case, the first ultrasound showed a normal aorta, but a large pseudoaneurysm was detected one month later.

Standardized diagnosis of primary fistulas, as well as the management of such patients, is especially difficult^10^ because primary fistulas are not frequent (Table 1). When the primary fistula has an etiology other than an aneurysm, such as aortitis,^4-8^ or when it is idiopathic, ^16-19^ diagnosis difficulties increase. For two-thirds of the patients, the diagnosis is made in the operating room^15^ (Table 2). The classic trio of abdominal pain, palpable mass and gastrointestinal bleeding only occurs in 6%to 12% of patients.^3^^,^^4^

**Table 1:** Samples of patients affected by primary aortoenteric fistula from different literature reviews, with number of operated patients and surgical results

Literature review	Number of cases reviewed	Number of patients operated	Patients that survived	Mortality
Reckless et al.,*^11^* 1972	*131*	*20*	*8*	*60%*
Brenowitz et al.,^12^ 1976	100	20	10	50%
Reiner et al.,^13^ 1978	112	33	15	55%
Daugherty et al.,^14^ 1979	49	25	14	44%
Sweeney et al.,^1^ 1984	118	33	21	36%
Calligaro et al.,^4^ 1992	226	82	44	46%
Dossa et al.,^15^ 1994	65	57	36	37%
Voorhoeve et al.,^3^ 1996	243	54 (starting from 1984)	29	46%

**Table 2:** Characteristics of patients with primary aortoenteric fistula due to aortitis without previous aortic aneurysm

Authors/year	Gender	Age	Clinical findings	Diagnostic approach/fistula site	Etiological agent	Treatment	Outcome
McIntyre et al.,^6^ 1981	M	73	Diabetes, low back pain, fever, pulsatile mass	Laparotomy: *3^rd^ portion of duodenum*	Arizona hinshawii	*Aortic division + axillo- bifemoral bypass*	*Satisfactory after 9 months*
Goldbaum et al.,^7^ 1986	M	75	Abdominal pain, fever, hematemesis	Laparotomy: *3^rd^ portion of duodenum*	Mycobacterium tuberculosis	*Dacron graft* in situ	*Satisfactory after 20 years*
Morrow et al.,^8^ 1987	F	32	Abdominal pain, back pain, hematemesis	Laparotomy: *4^th^ portion of duodenum*	Salmonella enteritidis	*Dacron graft* in situ	*Satisfactory after 3 years*
Wheeler et al.,^5^ 1992	M	63	Melena, abdominal pain, pulsatile mass	Laparotomy: *4^th^ portion of duodenum*	Mycobacterium tuberculosis	*Dacron graft* in situ	*Satisfactory after 7 years*
Calligaro et al.,^6^ 1992	F	60	Abdominal pain, fever	Laparotomy: *3^rd^ portion of duodenum*	Streptococcus viridans	*Aortic division*	*Death in the operating room*

With regard to aortoenteric fistula, hematemesis and melena form the most common symptoms (32% to 78%).^13,20^ When the etiology is an aortic aneurysm, a palpable mass can be found in 25% to 70% of the patients.^1,4,13^

Endoscopy is essential. However, it has the potential risk of inducing massive hemorrhage by dislodging fresh thrombus in the fistula.^16,17^ In our case, the endoscopy was repeated in order to achieve a diagnosis. We believed that making the patient undergo a laparotomy without diagnosis would be hazardous. Rarely can angiography demonstrate the fistula, as the bleeding is usually not active at the time of the examination.^16^^,^^18^

The outcome will depend upon the timeliness of diagnosis, the patient's general state, the degree of contamination, and the anatomical site of the aorta involved. The conventional treatment of infrarenal aortic infection includes primary intestinal suture or resection and intestinal anastomosis, excision and drainage of infection with the oversewing of the infrarenal aorta, combined with axillofemoral bypass grafting.^21^ The alternative of extraanatomical grafting is used in situations where the above cannot be performed, i.e. in infectious aneurysms of the aorta that involve the visceral branches.^22^ In these cases, the synthetic prosthesis is placed *in situ.* In the infrarenal aortic segment, in the absence of gross pus at the site of the fistula, *in situ* prosthesis grafting could be performed.^23^^,^^24^

Alternative reconstruction methods have been proposed and consist of *in situ* replacement with an antibiotic-bonded prosthesis, ^25^ homografts,^26^ and reconstruction with femoral veins.^27^ Additional maneuvers to prevent prosthesis infection include the use of viable pedicles of the greater omentum between aortic grafts and intestinal suture,^23^ and prolonged antibiotic therapy.^24^ In our case, the option was for extra-anatomical reconstruction, owing to the high risk and difficulty of carrying out *in situ* prosthesis placement on an infected, friable aorta.

Early diagnosis and aggressive surgical treatment are the best ways to achieve successful results in aorta-infected patients. The multifactorial features of this condition rule out one single approach, and the medical team must have knowledge of several forms for its presentation, as well as several options for dealing with this malady.
